# Immune Checkpoint Molecules: A Review on Pathways and Immunotherapy Implications

**DOI:** 10.1002/iid3.70196

**Published:** 2025-04-17

**Authors:** Erfan Rezazadeh‐Gavgani, Reza Majidazar, Parisa Lotfinejad, Tohid Kazemi, Ali Shamekh

**Affiliations:** ^1^ Student Research Committee Tabriz University of Medical Sciences Tabriz Iran; ^2^ Immunology Research Center Tabriz University of Medical Sciences Tabriz Iran; ^3^ Department of Immunology Tabriz University of Medical Sciences Tabriz Iran; ^4^ Aging Research Institute Tabriz University of Medical Sciences Tabriz Iran

**Keywords:** CTLA‐4, immune checkpoint inhibitor (ICI), immune modulators, immunotherapy, PD‐1, PD‐L1

## Abstract

**Background:**

Today, treating cancer patients with monoclonal antibodies (mAbs), by targeting immune checkpoints, is one of the most outstanding immunotherapeutic methods. Immune checkpoints are special molecules having regulatory role in immune system responses. Once these molecules are presented on cancer cells, these cells will be capable of evading the immune system through their own specific pathways. This Evasion can be prevented by counterbalancing immune system responses with immune checkpoints related antibodies.

**Aims:**

The current study aimed to highlight immunotherapy and its methods, describe the immune checkpoints pathways, outline the immune checkpoint inhibitors (ICIs), and recent advances in this field, and sketch an outlook on the best treatment options for the most prevalent cancers.

**Materials & Methods:**

This research implemented a narrative review method. A comprehensive literature review on the history, molecular and cellular biology, and the clinical aspects of immune checkpoint molecules was performed to illustrate the pathways involved in various cancers. Also, currently‐available and future potential immunotherapies targeting these pathways were extracted from the searched studies.

**Results:**

The immune checkpoint family consists of many molecules, including CTLA‐4, PD‐1, PD‐L1, LAG‐3, TIM‐3, and TIGIT. Attempts to modify these molecules in cancer treatment led to the development of therapeutic monoclonal antibodies. Most of these antibodies have entered clinical studies and some of them have been approved by the Food and Drug Administration (FDA) to be used in cancer patients' treatment plans.

**Discussion:**

With these novel treatments and the combination therapies they offer, there is also hope for better treatment outcomes for the previously untreatable metastatic cancers. In spite of the beneficial aspects of immune checkpoint therapy, similar to other treatments, they may cause side effects in some patients. Therefore, more studies are needed to reduce the probable side effects and uncover their underlying mechanism.

**Conclusion:**

Based on the data shown in this review, there is still a lack of knowledge about the complete properties of ICIs and the possible combination therapies that we may be able to implement to achieve a better treatment response in cancer patients.

## Graphical abstract

Immune checkpoint molecules are today's leading targets for immunotherapy, especially in the field of cancer. This review summarizes the most important and recent advances in this field and in different types of cancer.

## 1 Introduction

In contrast to conventional cancer therapies such as chemotherapy and radiotherapy, immunotherapy provides a novel approach that dynamically regulates the immune system to fight tumor cells in several targets and ways [1]. Immunotherapy is primarily applied to enhance the immune system through modifying the immunological microenvironment, allowing immune cells to target and eliminate tumor cells at numerous key points [2].

Programmed cell death protein 1 (PD‐1), cytotoxic T‐lymphocyte‐associated protein 4 (CTLA‐4), lymphocyte‐activation gene 3 (LAG3), T‐cell immunoglobulin mucin‐3 (TIM‐3), T‐cell immunoglobulin and ITIM domain (TIGIT), and B and T lymphocyte attenuator (BTLA) are just a few of the inhibitory immunoreceptors that have already been discovered and researched in cancer throughout the recent years. They are molecules which operate as the gatekeepers of the immune responses and are thus referred to as “immune checkpoints” [3]. Antibodies that hinder ligand‐receptor interaction may substantially reduce immunoreceptor functions. Anti‐PD‐1/PD‐L1 treatment is currently the most effective immune checkpoint inhibitor (ICI), and it has been authorized to treat a broad range of malignancies, including blood, skin, lung, liver, bladder, and kidney cancers. Immune checkpoint blocking treatment produces a longer‐lasting response compared to chemotherapies or other targeted therapies, possibly due to the immune system's memory. The poor immune checkpoint inhibition treatment response rate in most malignancies, ranging from 10% to 30%, is a substantial obstacle [4]. To create next‐generation medicines and enhance clinical guidelines for present regimens, a better knowledge of checkpoint pathways' biology is required now more than ever.

Previous reviews, such as a review by Nirschl et al. [5], have underlined the importance of the immune checkpoint molecules and pathways, but since the knowledge on this field is rapidly growing, an updated approach is required to investigate the applicability of these pathways into practice. This review delves deeply into the biological and clinical aspects of immunotherapy, and evaluates the evidence on tumor immunity to address the current advances in ICIs. It also provides timely clinical knowledge on the currently available targeted therapies, approved and suggested, for various cancers, reporting their known pros and cons alongside their outcomes. The broader, updated, and more clinically‐focused approach of this work are the key distinguishing factors compared to the previously published reviews.

## 2 Immune Checkpoint

The immune system must be able to discriminate between self and non‐self‐cells to respond in the right way. Any cells lacking host‐specific recognition patterns, can potentially induce immune responses [6]. After an appropriate response, an intact immune system limits its response to prevent any adverse events [7]. T‐cell immune response activation is a result of two signals, one from TCR and the other one from co‐stimulatory pathways [6]. The signals coming from co‐stimulatory pathways have the role of amplifying the response, and the absence of them may result in anergy, which is the irresponsiveness of immune cells to a trigger. The immune response will get attenuated and finish in many ways, including the inhibitory pathways (e.g., CD28 and PD‐1 pathways) [8, 9, 10, 11, 12]. The PD‐1 and CTLA‐4 are associated with the CD28 pathway, diminishing the immune responses [13]. Binding of PD‐1 to PD‐L1 downregulates the immune responses [14]. This interaction results in the suppression of T lymphocytes, and the reduction of cellular metabolism [13]. On the other hand, CTLA‐4 is a cell‐surface receptor that binds to its corresponding ligands like B7.1 and B7.2, as well as making them less available for CD28; it thus limits the activity of T lymphocytes and suppresses the immune response [11, 15, 16].

Recent studies on the immune checkpoints have resulted in the recognition of other inhibitory molecules, including LAG‐3, TIM‐3, and TIGIT [17, 18, 19].

It is believed that LAG‐3 expression on CD4+ and CD8+ T cells could trigger T cell exhaustion [17, 20]. Moreover, TIM‐3 and TIGIT mediate some downregulatory processes in tumor microenvironment, which result in the inhibition of T cells [18, 21, 22, 23].

### 2.1 PD‐1

PD‐1 belongs to the immunoglobulin (Ig) superfamily [24] and is composed of 288 amino acids [25]. This molecule is a Type I transmembrane glycoprotein consisting of a membrane‐permeating domain, an extracellular domain, and a cytoplasmic tail [26]. The cytoplasmic domain has an N‐terminal sequence forming an immunoreceptor tyrosine‐based inhibitory motif (ITIM) and a C‐terminal sequence, forming an immunoreceptor tyrosine‐based switch motif (ITSM) [24, 27]. Src homology 2 domain tyrosine phosphatases 1 (SHP1) and 2 (SHP2) are able to bind to ITSM [27]. The extracellular domain of PD‐1 shares about 21%–22% identical sequences with the other members of the CD28 family [26]. PD‐L1 and PD‐L2 also show 37% identical sequences [28].

First, the expression of PD‐1 occurs during thymic development. More precisely, TCR‐β rearrangement brings about PD‐1 expression in CD4^−^ CD8^−^ T cells [29, 30]. PD‐1 generally appears on immune cells including natural killer T (NKT) cells, B lymphocytes, dendritic cells (DCs), induced monocytes, and mature T cells (CD4^+^ and CD8^+^). PD‐1 expression is triggered by many factors such as antigen receptor signaling (TCR or B‐cell receptor signaling), cytokines, interleukin‐2 (IL‐2), IL‐7, IL‐15, IL‐21, infectious agents, and lipopolysaccharide (LPS) [31, 32].

The PI3K/AKT signaling pathway participates in many cellular processes such as proliferation and apoptosis. This signaling pathway is the first target of the suppressive function of PD‐1 [33]. During T cell activation, casein kinase 2 (CK2) phosphorylates PTEN, resulting in PTEN phosphatase activity suppression. PTEN dephosphorylates phosphatidylinositol 3,4,5 triphosphate (PIP3) at the 3′ position to generate phosphatidylinositol 4,5‐bisphosphate (PIP2), making PIP3 less available in the cell. Consequently, PTEN directly opposes the activation of PI3K by reducing the concentration of PIP3. Therefore, T cell activation results in a reduction of PI3K inhibition. PD‐1 inhibits the phosphorylation of PTEN and increases PTEN phosphatase activity by inhibiting CK2, terminating PI3K/Akt pathway's initiation [31, 33]. Consequently, cellular proliferation is decreased [13].

**Figure 1:**
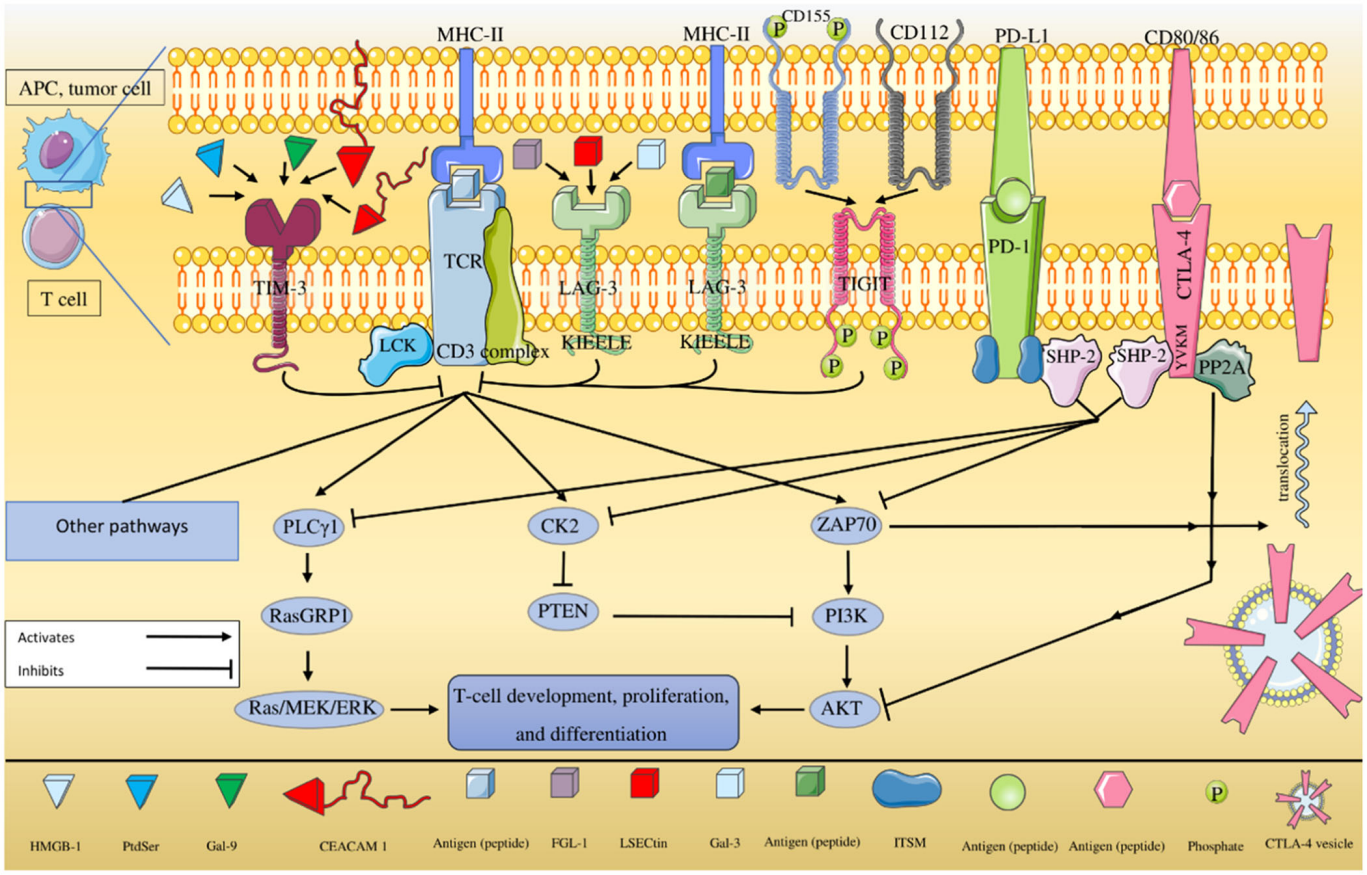
Inhibition of T‐cell activation. In T cell activation, CK2 phosphorylates PTEN. PTEN inhibits PI3K, which leads to the dephosphorylation of PIP3 to generate PIP2, making PIP3 less available in the cell. Therefore, T cell activation results in a reduction of PI3K inhibition. PD‐1 inhibits the phosphorylation of PTEN and stimulates PTEN phosphatase activity by inhibiting CK2, thus terminates PI3K/Akt pathway initiation. Therefore, T cell proliferation will decrease. SHP‐2 binds to the cytoplasmic tail of PD‐1 from the site of ITSM. Another signaling pathway that PD‐1 affects is the Ras/MEK/ERK pathway. MEK‐ERK pathway is downstream to Ras, and Ras signaling pathway is important for T cell development, proliferation, and differentiation. Ras includes diacylglycerol (DAG)‐binding domain and a couple of calcium‐binding EF‐hands. PLCγ1 hydrolyzes PIP2, resulting in DAG and IP3 generation. IP3 stimulates release of calcium from intracellular stores. DAG and Ca^2+^ are necessary for the activation of RasGRP1. Hence, PLCγ1 activation brings about RasGRP1 activation. PD‐1 has the ability of PLCγ1 inhibition, and actually, PD‐1 inhibits TCR linkage to Ras and restricts T cell development, proliferation, and differentiation. Parts of this figure were created using the freely available samples on https://smart.servier.com/; the full figure is an original take from the authors.

The second signaling pathway that PD‐1 affects is the Ras/MEK/ERK pathway. MEK‐ERK pathway is downstream to Ras. On the other hand Ras signaling pathway is important for T cell development, proliferation, and differentiation. RAS guanyl nucleotide‐releasing protein (RasGRP) has a role in linking TCR signaling to Ras, and includes diacylglycerol (DAG)‐binding domain and a couple of calcium‐binding EF‐hands. PLCγ1 hydrolyzes PIP2, resulting in DAG and inositol 1,4,5‐trisphosphate (IP3) generation. IP3 brings Ca^2+^ inflow and stimulates the release of calcium from intracellular stores [34, 35, 36, 37]. DAG and Ca^2+^ are necessary for the activation of RasGRP1. Hence, the activation of PLCγ1 brings about RasGRP1 activation. PD‐1 can inhibit PLCγ1; as a matter of fact, PD‐1 inhibits TCR linkage to Ras, restricting T cell development, proliferation, and differentiation. PD‐1 can suppress other pathways, as well as the Zeta chain of T cell receptor‐associated protein kinase 70 (ZAP70) associated pathways [31, 38] (Figure 1).

### 2.2 CTLA‐4

The CTLA‐4 gene consists of 4 exons [39] and is located on q33 band of chromosome 2 (2q33) [40]. Full‐length CTLA‐4 (flCTLA‐4) is the result of 4 exon expression and is made up of a leader peptide, the ligand‐binding domain or IgV‐like domain, the transmembrane domain, and the cytoplasmic domain [40, 41]. After the crosslinking of CD3 and CD28 on T lymphocytes, the expression of flCTLA4 is induced [42, 43]. CTLA‐4 is undetectable on naive T cell [44], but its expression is upregulated after 2–3 days of T cell activation [45]. Several factors and multiple mechanisms affect the CTLA‐4 expression such as nuclear factor of activated T‐cells (NFAT), Foxp3, and microRNAs (miRNAs) [12]. The expression and cellular position of CTLA‐4 is strongly modulated. Its expression relies on IL‐2 receptor‐ and CD28‐mediated signals [46].

It has been shown that CTLA‐4 plays its role as an immune checkpoint molecule in many ways [35, 47, 48]. CTLA‐4 takes part in T cell inhibition by intracellular and extracellular functions. The intracellular functions include delivering cell‐intrinsic negative signal (inhibitory signaling), ligand‐independent inhibition, ligand competition, and affecting adhesion and motility. On the other hand, the extracellular functions include activating indoleamine 2,3‑dioxygenase (IDO), inducting inhibitory cytokines, diminishing CD28 ligands’ (CD80 and CD86) availability, and internalizing CD28 ligands in antigen‐presenting cells (APCs). All of the above issues have been reviewed by Walker et al. [49]; but in the present study, some of these are further elaborated.

Both CD28 and CTLA‐4 can bind to CD80 (B7.1) and CD86 (B7.2) on T cells, but with different affinities. Higher affinity of CTLA‐4 makes its binding to B7 family easier than CD28, which brings about the B7 family less available for CD28. This results in the attenuation of CD28 co‐stimulatory pathway [48]. Signals coming from the binding of CD28 to the B7 family are important to avoid T cell non‐responsiveness, anergy, and cell death. For instance, the most important function mediated by the CD28 co‐stimulatory pathway is IL‐2 production, which is important for T‐cell proliferation and differentiation into an effector cell. CTLA‐4 is initially placed in intracellular components; for instance, endosomes and lysosomes. Thus, the detectable amount of CTLA‐4 on the cell surface is primarily low, and higher ligation affinity is focused predominantly on the intracellular sites [48, 50, 51]. There is an interaction between the clathrin adaptor complex AP‐2 and the tyrosine‐based motifs in the cytoplasmic domain of CTLA‐4, resulting in the internalization of CTLA‐4. Phosphorylation in this motif disrupts the interaction with AP‐2, after which, CTLA‐4 is stabilized on the cell surface [52].

CTLA‐4 can bind to PI3K, and it has been indicated that CD28 and CTLA‐4 share a signaling function in T cells [53]. The tail of CTLA‐4 contains potential motifs that interact with proteins, and consequently, interfere with TCR and CD28 signaling [54]. Also, CTLA‐4 can inhibit early TCR signaling events within the TCR complex [55].

The key role of ZAP70 in T‐cell development and activation has also been indicated in previous studies [56]. After the interaction of TCR with major histocompatibility complex (MHC) class II on APCs, coreceptor‐associated Lck is transported into the vicinity of CD3 complex. After this location change, Lck phosphorylates immunoreceptor tyrosine‐based activation motifs (ITAMs). Phosphorylation of ITAMs results in ZAP70 recruitment, which in turn contributes to PI3K pathway activation [57, 58, 59, 60, 61]. CTLA‐4 can reduce ZAP70 phosphorylation and fulfill T cell suppression [62] (Figure 1).

SHP‐2 can initiate the RAS pathway, and protein phosphatase 2A (PP2A) inhibits the phosphorylation of Akt; both of these molecules need the YVKM motif located in the intracellular domain of CTLA‐4 for this association [63].

### 2.3 Lymphocyte Activation Gene‐3 (LAG‐3)

LAG‐3, also known as CD223, is a recently discovered inhibitory immune‐checkpoint. It is comprised of four domains (D1–D4) and is a member of type I transmembrane proteins [64, 65]. Of three regions of cytoplasmic domain of LAG‐3, a motif, known as KIEELE motif, is essential for LAG‐3 inhibitory effects. The gene responsible for coding LAG‐3 is located on chromosome 12 in humans [17].

It has been reported that some molecules, including IL‐2, IL‐7, interferon gamma, NFAT, thymocyte selection‐associated high mobility group box protein (TOX), and nuclear receptor subfamily 4 (NR4A) could augment the expression of LAG‐3 on activated T cells [17, 65].

It is believed that consistent antigen stimulation can induce LAG‐3 expression on tumor‐infiltrating CD4^+^ and CD8^+^ T cells. It is worth noting that in patients with colorectal cancer (CRC) and melanoma, peripheral blood regulatory T cells (T_reg_) express LAG‐3. Other cells such as natural killer (NK) cells and plasmacytoid dendritic cells also express LAG‐3. The negative regulatory effect of LAG‐3 on T cell receptor signal transduction in cooperation with other inhibitory immune‐checkpoints could lead to the exhaustion of T cells. The exact LAG‐3 mechanism of action is yet to be elucidated [17, 20].

In tumor microenvironment, dampening of T cell functionality and T cell exhaustion results in tumor progression and tumor evasion from the immune system. It has been shown that the inhibitory effects of LAG‐3 are strongly correlated with its level of expression on T cells [17, 20, 64].

Due to the structural similarities between LAG‐3 and cell surface CD4, LAG‐3 is able to bind to MHC‐II [17]. Moreover, Galectin‐3 (Gal‐3), fibrinogen like protein 1 (FGL‐1), and liver sinusoidal endothelial cell lectin (LSECtin, CLEC4G) are other ligands interacting with LAG‐3. Recent researches suggest that LAG‐3 is a synergistic partner to PD‐1 in suppressing antitumor immunity [20, 64].

Higher levels of LAG‐3 are associated with unfavorable prognosis and poor clinical outcome in different types of solid tumors and hematologic malignancies; including renal cell carcinoma, bladder cancer, breast cancer, CRC, hepatocellular carcinoma (HCC), head and neck squamous cell carcinoma, chronic lymphocytic leukemia, diffuse large B cell lymphoma, and follicular lymphoma. Furthermore, it has been reported that higher levels of LAG‐3 may correlate to favorable outcomes in some patients with esophagus adenocarcinoma and gastric cancer who have undergone an anti‐PD‐1 antibody treatment [20, 65, 66].

### 2.4 T‐Cell Immunoglobulin Domain and Mucin Domain‐3 (TIM‐3)

TIM‐3, a Type‐I transmembranous protein, is an immune‐checkpoint with regulatory effects on both the innate and adaptive immune systems. This immunoregulatory molecule is mainly expressed on monocytes, dendritic cells, NK cells, and effector T cells. Chronic antigen stimulation is the canonical factor in persistent TIM‐3 expression on aforementioned cells. The gene responsible for TIM‐3 coding is located at 5q33.2 in the human genome [18, 64].

Some established ligands of TIM‐3 are Gal‐9, high‐mobility group box‐1 (HMGB‐1), phosphatidylserine (PtdSer), and carcinoembryonic antigen‐related cell adhesion molecule‐1 (CEACAM‐1) [18]. It has been proposed that Gal‐9 alters Th1 or Th17 responses through regulating IL‐12 and IL‐13 production [67].

Recent in vitro and in vivo studies have illustrated that IL‐12, IL‐27 and IFN‐beta are among the factors that augment TIM‐3 expression [18, 21, 64, 67]. The inhibition of Th1 responses; and changing the expression of TNF and interferon gamma are prominent effects of TIM‐3 as an immunomodulator. As TIM‐3 is expressed on tumor‐infiltrating dendritic cells, innate anti‐tumor responses are dampened. Details of the signaling pathway mechanisms of TIM‐3 are still unknown [21, 67].

Studies have revealed that TIM‐3 is associated with poor clinical outcome in some hematopoietic malignancies; including myelodysplastic syndrome (MDS) and acute myeloid leukemia [18, 68]. Unfavorable prognosis of solid tumors, including HBV‐induced HCC, non‐small cell lung cancer (NSCLC), colon cancer, urothelial carcinoma, gastric cancer, cervical cancer, and clear cell renal cell carcinoma, may be associated with higher levels of TIM‐3 [21].

### 2.5 T Cell Immunoglobulin and ITIM Domain (TIGIT)

TIGIT, a member of the Ig superfamily, is a recently discovered co‐inhibitory agent on immune cells. TIGIT is expressed on some subtypes of T cells (effector, memory and regulatory) and NK cells [23, 64].

It has been elucidated that CD155 (previously known as poliovirus receptor; i.e., PVR), CD112 (nectin‐2 or PVR‐related protein 2), and CD113 (nectin‐3 or PVR‐related protein‐3) are among major ligands of TIGIT [19, 69]. The pathway in which TIGIT is involved, negatively affects TCR signaling [64]. Alterations in TCR signaling diminishes the activation and proliferation of T cells. Besides, TIGIT pathway promotes the expression of some anti‐apoptotic agents, including IL‐2, IL‐15, and Bcl‐xL. The aforementioned factors together lead to the functional inhibition of T cells [23, 64, 70].

TIGIT tends to enhance Th2 cytokines alongside IL‐10; it also inhibits interferon gamma and IL‐17 production. There are evidence suggesting that TIGIT synergizes with PD‐1 and impairs anti‐tumor responses in cooperation with TIM‐3 [19, 22].

It is proposed that TIGIT may take part in tumor progression in some solid tumors and hematologic malignancies, including refractory multiple myeloma, metastatic NSCLC, esophageal cancer, cervical cancer, and melanoma [19, 22, 70].

## 3 Immunotherapy

Immunotherapy alters the immune system to treat cancer(s). It is a type of biological therapies used for treating cancers and includes different methods such as ICIs, vaccines, and cytokines [71, 72]. In this article, we have focused on ICIs and their applicability in various cancers, providing their currently known outcomes. This review also summarizes the side effects and the treatment of choices in different cancer settings, illustrating an updated clinical knowledge for practitioners.

### 3.1 Cancer Treatment Vaccines

Although vaccines are usually known as immunoprophylactic agents, such as Gardasil for human papillomavirus (HPV) induced cervical cancer prevention, some vaccines may also be used for immunotherapy. These vaccines, including Sipuleucel‐T and Talimogene laherparepvec (T‐VEC), have been shown to be effective in prostate cancer and melanoma, respectively [73, 74, 75, 76, 77].

### 3.2 Adjuvant Therapy

Immunotherapy with BCG was first reported to be effective for bladder cancer treatment in 1976 [78]. Nowadays, it is the choice treatment for non‐muscle‐invasive bladder cancer. Although the mechanism of antitumor effect of BCG is still unclear, and we cannot definitely determine whether the response is antigen specific or non‐antigen specific, its mechanism is more likely to be antigen specific [79].

### 3.3 Cytokines

Granulocyte‐macrophage colony‐stimulating factor (GM‐CSF) and granulocyte colony‐stimulating factor (G‐CSF) are cytokines that regulate cellular maturation in the bone marrow. Therefore, utilizing these cytokines can affect T‐cell production; and as a matter of fact, these cytokines are useful for treating various cancers [80]. Sargramostim, a recombinant human GM‐CSF, is proposed by Lazarus et al. [81] as an adjuvant in anti‐cancer immunotherapy.

IL‐2 has a role in proliferation and activation of T‐cells, and it can consequently be used for treating cancers. Aldesleukin is a recombinant IL‐2, which has been approved by the FDA for the treatment of metastatic melanoma [82]. As reviewed by Kirkwood et al. [83], other cytokines can also be used for immunotherapy, including interferon alfa (IFN‐α).

### 3.4 T‐Cell Transfer Therapy

Adoptive T‐cell transfer (ACT) can be used to treat certain tumors, viral infections, and inflammatory diseases. There are three forms of ACT developed for cancer therapy, including tumor‐infiltrating lymphocytes (TILs), T‐cell receptor (TCR) engineered T‐cells, and chimeric antigen receptor (CAR) T‐cells. Many clinical trials have suggested that TILs can induce durable effects on metastatic melanoma [84, 85]. The activation of T‐cells is a result of TCR recognizing peptides presented on MHC complexes; this limitation makes tumor cells capable of avoiding the immune system by MHC‐associated antigen loss [84]. However, CARs can recognize unprocessed antigens on tumor cell surface, such as carbohydrate or glycolipid structures, without incorporating MHC. Therefore, CAR T‐cells can overcome tumor cell immuno‐evasion. “Tisagenlecleucel” (KYMRIAH) is the first FDA approved drug with the technology of CAR in the drug market. The second drug was “axicabtagene ciloleucel” (YESCARTA) with a lower cost; but both drugs are still considered to be expensive [84, 85, 86].

### 3.5 Monoclonal Antibodies (mAbs)

B lymphocytes are capable of identifying antigens and producing antibodies. Antibodies bind to the epitope regions on the antigens. If the immune response produces an antibody recognizing just one epitope of the antigen, it will be called a mAb.

Nowadays, mAbs are the most commonly used and approved agents in cancer immunotherapy. The main cancers treated with mAbs include breast cancer, colon cancer, and lymphoma. The common side effects of using mAb include fever, fatigue, headache, bleeding, and other unspecific signs and symptoms. Mechanism of action differs among mAbs; but despite various mechanisms, they are used as a part of standard treatment procedure besides chemotherapy and/or radiotherapy [86]. The mAbs have different targets. For instance, Rituximab, Trastuzumab, and Ipilimumab are among well‐known mAbs, and their targets are CD20, human epidermal growth factor receptor 2 (HER2), and CTLA‐4, respectively [87].

One of the ways that tumors implement to avoid the immune system is utilizing immune checkpoint molecules. As mentioned before, immune checkpoints can suppress T‐cells; hence, we can abolish immune evasion by immune checkpoint blockade. The mAbs used for this goal are ICIs, targeting immune checkpoint molecules. Among immune checkpoint molecules, CTLA‐4 and PD‐1/PD‐L1 have been more examined as mAb targets; however, in the recent years, other molecules, such as lymphocyte activation gene 3 protein (LAG‑3), T‐cell Ig, and mucin domain containing‐3 (TIM‐3) are being more evaluated with the hope of achieving better therapeutic effects and fewer side effects [88, 89, 90].

### 3.6 Bispecific (bsAb) Antibodies

Although natural antibodies primarily target only one specific antigen; a bsAb is a synthetic molecule that can bind to two distinct kinds of antigens, or two distinct epitopes on the same antigen at the same time. These antibodies can be used for a range of diagnostic and therapeutic purposes. BsAbs can be utilized in combination with horseradish peroxidase (HRPO), in pre‐targeting techniques, and to improve imaging for early diagnosis. BsAbs can also target immune cells specifically, aid and respond to immune cells, fine‐tune the destiny and functionality of immune cells, increase immune cell tolerance, and facilitate immunological homeostasis in the treatment of cancer. Other disorders in which the BsAbs can be used, include hemophilia A, diabetes, Alzeimer's disease, and ophthalmological disorders [91]. BsAbs are nowadays often being investigated for medication delivery and cancer immunotherapy; but they can also be used to treat various disorders [91, 92]. These antibodies are available in a variety of forms, but “IgG‐like” and “Non‐LgG‐like” antibodies are the two most common [92].

#### 3.6.1 IgG‐Like

Similar to the traditional format of mAbs, this format of bsAb consists of Fab arms and an Fc region; but unlikely, these two Fab arms bind to different antigens. Trifunctional antibodies are the most prevalent kinds, as they contain three distinct binding sites on the antibody: two on the Fab domains and another on the Fc domain [93, 94].

#### 3.6.2 Non‐IgG‐Like

This format of bsAb lacks the Fc domain, and only includes two Fab arms (Fab and Fab') [91]. The bi‐specific T‐cell engagers (BiTEs) are the most evolved forms of these antibodies [95]. The production of chemically‐linked Fabs is expensive compared to BiTEs, despite working in an identical way [96]. After clinical studies, the FDA approved tebentafusp as a BiTE for the treatment of HLA‐A*02:01‐positive adult patients with unresectable or metastatic uveal melanoma in January 2022 [97].

In comparison to traditional mAb, bsAbs have the capacity to activate T‐cells. The Fab regions in mAb will be utilized to attach tumor cells, while T‐cell lacks Fc receptors. In contrast, bsAbs use the other Fab domain (Fab') to bind to the T‐cell [98]. Having a higher cytotoxicity potency is the second advantage of bsAbs as they can bind to poorly expressed antigens [99]. Non‐lgG‐like bsAbs potentially reach some antigens that regularly are unreachable for traditional mAbs; as these bsAbs are smaller in size, compared to traditional mAbs [92].

The ability to target more than one specific molecule at the same time, can help minimize resistance to therapy, as they can modulate redundant pathways. Since many diseases have numerous multidimensional impacts throughout the body, binding or inhibiting several targets in a pathway can be helpful in controlling diseases [100]. IgG‐like bsAbs interact with immune cells by the employment of Fc domains; thus they can be immunogenic [92].

## 4 The mAbs and Cancer

### 4.1 Radioimmunotherapy (RIT)

The use of radioactively conjugated murine antibodies targeting cell antigens is known as RIT. Murine antibodies have been chosen to reduce the radiation dose because of their strong immunogenicity, which accelerates fast disease elimination. Since lymphomas are extremely radio‐sensitive cancers, the majority of studies have focused on their use in lymphomas [101, 102]. Tositumomab is a CD20‐targeting mAb that was used to treat non‐Hodgkin's lymphoma in combination with iodine‐131 in years 2003–2014 [103].

### 4.2 Antibody‐Directed Enzyme Prodrug Therapy (ADEPT)

Cancer‐associated mAbs conjugated to a drug‐activating enzyme are used in this procedure. Despite the fact that ADEPT is a strategy for dealing with tumor selectivity difficulties, they have a low clinical success rate [104, 105].

### 4.3 Antibody‐Drug Conjugates (ADCs)

Antibodies that are conjugated to one or more therapeutic agents are known as ADCs. ADCs are a type of biopharmaceutical agents that are used to treat cancer as a targeted therapy. ADCs integrate the cancer‐killing capacity of cytotoxic agents with the targeting capabilities of mAbs. As a matter of fact and unlike chemotherapy, they are designed to specifically attack and destroy tumor cells while leaving healthy cells intact [106, 107, 108].

### 4.4 Immunoliposome Therapy

Antibody‐conjugated liposomes are known as immunoliposomes. Liposomes have the potential to transport drugs or therapeutic nucleotides; and when combined with mAbs, they can be used to target cancerous cells [109]. By using an antibody fragment against the human transferrin receptor, immunoliposomes have been effectively utilized in vivo to carry tumor‐suppressing genes into the tumor site. Tissue‐specific gene transfer was performed through utilizing immunoliposomes in brain and breast cancer [110].

### 4.5 Checkpoint Therapy

As discussed above, mAbs are categorized into several groups including anti‐CTLA‐4, anti‐PD‐1, anti‐LAG3, and other groups (Table 1). This study mainly focuses on cancer monotherapy and combination therapy using anti‐CTLA‐4/PD‐1/PD‐L1 [72, 111]. It is noteworthy to mention that in a recent cohort study by Gougis et al. [112], the use of immune checkpoint therapy in pregnancy was not associated with severe adverse neonatal or fetal events, which is a promising step in the use of these treatments. On the other hand, ICIs have been shown to increase coronary calcium deposition, increasing the possibility of adverse cardiovascular events [113]. These findings necessitate further investigations in the field of ICIs.

**Table 1:** ICIs in cancer immunotherapy.

Antibody	Target	Ig type	Approved condition	Undergoing trials	Side effects	References
Ipilimumab	CTLA‐4	IgG1	Metastatic melanoma, dMMR colorectal cancer, and advanced RCC	NSCLC, SCLC, and prostate cancer	Colitis, hepatitis, dermatitis, neuropathies, endocrinopathies, pneumonitis, nephritis, and encephalitis	[47, 114, 115, 116, 117, 118, 119, 120]
Tremelimumab (Ticilimumab)	CTLA‐4	IgG2	Undergoing clinical trials	Metastatic urothelial cancer		[121, 122]
Nivolumab	PD‐1	IgG4	Metastatic melanoma, metastatic NSCLC, advanced RCC, classical Hodgkin lymphoma, Squamous cell carcinoma of the head and neck, urothelial carcinoma, dMMR colorectal cancer, and HCC	IDH mutated high‐grade glioma, recurrent dMMR prostate cancer, advanced cutaneous squamous cell carcinoma, and uterine cancer	Pneumonitis, colitis, hepatitis, endocrinopathies, nephritis, skin adverse reactions, and encephalitis	[47, 117, 119, 122, 123, 124]
Pembrolizumab	PD‐1	IgG4	Melanoma, NSCLC, head and neck cancer, and classical Hodgkin lymphoma	Metastatic HER2‐negative breast cancer, refractory esophageal cancer, and metastatic anal cancer	Fatigue, skin adverse reactions, arthralgia, pneumonitis, colitis hepatitis, endocrinopathies, and nephritis	[119, 124, 125, 126, 127]
Pidilizumab	PD‐1	IgG1	Undergoing clinical trials	—	—	[47, 128]
Toripalimab	PD‐1	IgG4	Undergoing clinical trials	Advanced biliary tract cancer, advanced HCC, small cell carcinoma of esophagus, and head and neck cancers	—	[129]
Atezolizumab	PD‐L1	IgG1	Locally advanced or metastatic urothelial carcinoma and metastatic NSCLC	Breast cancer, NSCLC, advanced thymus carcinoma, metastatic urothelial carcinoma, unresectable mesothelioma, and bladder cancer	Pneumonitis, hepatitis, colitis, and endocrinopathies	[119, 127, 130, 131, 132]
Avelumab	PD‐L1	IgG1	MCC	Neuroendocrine carcinoma, metastatic colorectal cancer, multiform glioblastoma, metastatic urothelial carcinoma	Colitis, pneumonitis, hepatitis, nephritis, and endocrinopathies	[119, 126, 133, 134, 135]
Durvalumab	PD‐L1	IgG1	Metastatic urothelial carcinoma	Non‐muscle invasive bladder cancer, HCC, NSCLC, and esophageal cancer	Hepatitis, pneumonitis, colitis, and endocrinopathies	[119, 127, 135, 136, 137, 138]
Dostarlimab‐gxly (JEMPERLI)	PD‐1	IgG4	dMMR recurrent or advanced endometrial cancer that has progressed on or following prior treatment with a platinum‐containing regimen and solid tumors that have progressed on or following prior treatment without satisfactory alternative treatment options	dMMR colorectal cancer, gestational trophoblastic neoplasia, recurrent/progressive cervix cancer, melanoma, lung cancer, and pancreatic cancer	Fatigue/asthenia, anemia, diarrhea, nausea, decreased lymphocytes/albumin/sodium, and increased alkaline phosphatase	[139, 140]

Abbreviations: dMMR, mismatch repair deficient; HCC, hepatocellular carcinoma; HER‐2, human epidermal growth factor receptor 2; IDH, isocitrate dehydrogenase; MCC, metastatic merkel cell carcinoma; NSCLC, non‐small cell lung carcinoma; RCC, renal cell carcinoma; SCLC, small cell lung cancer.

#### 4.5.1 CTLA‐4 as a Target

CTLA‐4 is the first target immune checkpoint molecule used in cancer treatment. Two well‐known mAbs used to target this molecule are ipilimumab and tremelimumab [87, 141]. Ipilimumab is a full human IgG1 antibody developed in 1999, which was then approved by the FDA in 2011 for treating unresectable metastatic melanoma [114, 115, 116]. A combination of some ICIs is also used in the treatment of malignancies. For example, combination of ipilimumab and nivolumab (an anti‐PD‐1 antibody) is approved in the treatment of advanced renal cell carcinoma and mismatch repair deficient (dMMR) CRC [117, 118, 142]. The development of cancer treatment strategies is still being pursued, and mAbs are being used in numerous clinical trials. For example, ipilimumab is being investigated to enter the treatment plans of non‐small cell lung carcinoma (NSCLC), small cell lung cancer (SCLC), and prostate cancer [116, 121].

Every therapeutic modality has its own specific side effects. Trials involving ipilimumab show that patients receiving this antibody may suffer some adverse effects such as pneumonitis, skin changes, nephritis, and hepatitis [116, 123].

The IgG2 variant of CTLA‐4 targeting antibodies is called tremelimumab, which is also a full human antibody. This agent is still under investigation and has not yet been approved [122].

The US national library of medical clinical trials information website shows more than 800 registered clinical trials around the world for ipilimumab. More than 200 of these trials have been completed up to now [143].

#### 4.5.2 Targeting PD‐1/PD‐L1

Searching for an alternative in cancer treatment led to the discovery of PD‐1 pathway's role in cancer development and its inhibitory antibodies. PD‐1 is another member of Ig superfamily, which can be targeted by various antibodies, including nivolumab, pembrolizumab, and pidilizumab [119].

Nivolumab is the most commonly used anti‐PD‐1 mAb in clinical trials [125, 128]. It has a full human IgG4 structure [47]. Metastatic melanoma, metastatic NSCLC, and HCC are some of nivolumab's approved indications [129]. Similar to ipilimumab, nivolumab is being investigated to be used in the treatment of malignancies such as the isocitrate dehydrogenase (IDH) mutated high‐grade glioma, advanced cutaneous squamous cell carcinoma (CSCC), and uterine cancer [130, 131]. The clinical trial of nivolumab in combination with radiotherapy for the treatment of advanced NSCLC is in progress [125].

It should also be noted that pneumonitis, encephalitis, and nephritis are some adverse reactions detected in patients undergoing treatment with nivolumab [119, 123, 125].

Pembrolizumab is a humanized IgG4 antibody developed after nivolumab to target PD‐1. Similar to nivolumab, pembrolizumab is approved to be used in the treatment of NSCLC patients. Classical Hodgkin lymphoma is the other indication of pembrolizumab [47].

Pembrolizumab is also being used in trials on metastatic anal cancer, refractory esophageal cancer, and metastatic HER2‐negative breast cancer patients [126, 133, 136, 144]. The effects of combination therapy with pembrolizumab and epacadostat in the treatment of thymic carcinoma are being investigated [137]. Fatigue, endocrinopathies, hepatitis, and pneumonitis are major adverse reactions of pembrolizumab [119, 123].

On 17th August 2021, another drug joined the FDA‐approved anti‐PD‐1 mAbs [145]. Dostarlimab‐gxly (JEMPERLI) granted accelerated approval for dMMR advanced solid tumors. In a clinical trial (NCT04165772), this medication treated CRC patients with 100% efficacy; but more research has to be done on this subject before it can be concluded whether or not this medicine is a great success.

Pidilizumab is an IgG1 mAb that binds to PD‐1 molecule. This brand new antibody shows promising outcomes in cancer treatment in recent clinical trials; however, it has not yet been approved to be used in the treatment of any conditions yet [47].

Atezolizumab, avelumab, and durvalumab are three antibodies developed to target PD‐L1, as an APC surface molecule [47]. These antibodies are IgG1 mAbs. Atezolizumab is a fully humanized antibody, but avelumab and durvalumab have a human source [47]. Atezolizumab and durvalumab have been approved by the FDA for the treatment of some certain types of urinary system carcinomas (Table 1) [132, 138]. Atezolizumab is also approved to be used in metastatic NSCLC patients [146]. Metastatic Merkel cell carcinoma (MCC) is the approved usage for avelumab [134]. Treatment response to combination therapy with atezolizumab and bevacizumab is currently being investigated in some clinical trials [147].

All three anti‐PD‐L1 antibodies described above are involved in multiple clinical trials of the urinary system malignancies, and the results are yet to come [146, 148, 149, 150, 151, 152]. Endocrinopathies, pneumonitis, and hepatitis are some of the commonly reported adverse reactions of these agents when used to treat cancer patients [123].

### 4.6 Treatments

#### 4.6.1 Breast Cancer

Breast cancer is the major aggressive malignancy in women globally [153]. This cancer, alongside lung cancer, are the most frequently detected malignancies, with 2.2 million cases recorded in 2020 [154]. Overall, 1 in 7 (14%) of women in the world is affected with breast cancer [155]. About 19%–50% of women with breast cancer will develop metastatic forms of this disease [156]. Breast cancer has the largest proportion of new cases (11.7%) worldwide and lung cancer (11.4%) and CRC (10.0%) take the second and third places in the ranking, respectively. Lung cancer was the most common cause of death from cancer (18%), followed by CRC (10%) [154, 157].

Trastuzumab is a mAb that targets HER2/neu and is the most well‐known antibody for treating breast cancer. This medicine has been investigated so comprehensively in recent years that systematic review studies on its usage in different conditions, even during pregnancy, have been conducted. According to a meta‐analysis research published in 2021, the likelihood of major adverse effects of trastuzumab in the early gestational stages of breast cancer patients under the age of 30 is essentially low [103].

Pembrolizumab's safety and tolerability in triple‐negative breast cancer (TNBC) patients has been demonstrated, and this medicine in conjunction with chemotherapy, has been found to enhance outcomes [158]. This means it can be used as a safer alternative to chemotherapy with fewer side effects.

The FDA has also authorized atezolizumab in combination with chemotherapy for PD‐L1^+^ advanced triple‐negative breast tumors [158].

It has been suggested that combining PD‐1/PD‐L1 suppression with neoadjuvant chemotherapy enhances the rate of pathological complete response in the initial phase of TNBC patients. There was no considerable increase in Grades 3–4 adverse events, serious adverse events, or treatment cessations in immunotherapy participants [159].

A systematic review assessing pembrolizumab, avelumab, and atezolizumab in metastatic TNBC patients has revealed that solo treatment with anti‐PD‐1/PD‐L1 agents shows promising outcomes, particularly when given early in the disease's progression; while combination methods with chemotherapy appear to improve the efficacy [160].

These data suggest that ICIs, especially PD‐1/PD‐L1 inhibitors, can be considered as new and approved strategies for breast cancer treatment.

#### 4.6.2 Non‐Small Cell Lung Cancer

Any kind of epithelial lung cancer, except small‐cell lung carcinoma, is referred to as NSCLC. NSCLC is responsible for around 85% of all lung malignancies [161]. The NSCLC survival rate has been reported to be 16.8% for men and 25.1% for women from 2012 to 2015, which is poor compared to other malignancies [162]. Depending on the data from people affected with NSCLC between 2011 and 2017, the 5‐year relative survival rate was 26% [163].

Landre et al. [164] have suggested that the first‐line treatment for advanced NSCLC (PD‐L1‐negative or lower than one percent expression of the PD‐L1‐positive type) with PD‐(L)1 inhibitors and chemotherapy combination regimen is better than monotherapy with chemotherapy, since this dual regimen meaningfully prolongs the overall survival and progression‐free survival. On the other hand, Liu et al. [165] believe that in terms of possible superior survival outcomes, combination treatment with PD‐1 inhibitors is superior to PD‐L1 inhibitors.

Furthermore, another study claims that using nivolumab in NSCLC patients’ management is not exactly expected to be effective in the real world, unlike clinical trials [166]. It is noteworthy that this study has included clinical trials of different types of NSCLC; thus their analyzed data might be heterogeneous.

It has been declared that in the case of advanced NSCLC (PD‐L1 positive type), anti‐PD‐(L)1 antibodies outperform chemotherapy in terms of survival outcomes and safety, while having adverse effects and being life‐threatening [167].

U. Dafni et al. [168] suggest that in the case of advanced NSCLC individuals’ treatment, chemotherapy combined with either pembrolizumab or atezolizumab regularly outperforms chemotherapy monotherapy or any other ICIs’ combination or monotherapy, notably in non‐squamous cases. They also concluded that a chemotherapeutic core is preferred over another ICI in combination therapies with another ICI.

The cost‐effectiveness of immunotherapy as a treatment approach to NSCLC in a variety of situations is elaborated in previous studies. Also the use of PD‐L1 expression as a biomarker or a modifying the price of these drugs has been suggested to further enhance the cost‐effectiveness of immunotherapy [169].

In NSCLC patients with at least 50% of PD‐L1 expression, monotherapy with ICI is likely to result in better overall survival rate, as well as a higher progression‐free survival and overall response rate compared to platinum‐based chemotherapy. It may also result in a lower frequency of adverse events and a better health‐related quality of life (HRQoL). Combination therapy with ICIs in these patients also possibly repeats the results in the overall survival rate; but due to the lack of evidence, its impact on progression‐free survival, overall response rate, and HRQoL is still unclear [170].

ICI treatment is seen to be effective as a first‐line treatment for patients suffering advanced NSCLC when paired with chemotherapy, and this is not dependent on PD‐L1 expression. In the patients suffering from advanced NSCLC with high levels of PD‐L1 expression, monotherapy can also be beneficial as a first‐line treatment. When ICI is used as a second‐line therapy, its effectiveness and safety exceeds docetaxel [171].

A study evaluating the efficacy of PD‐(L)1 inhibitors accompanying chemotherapy has evaluated whether lung cancer cases may benefit from the treatment even with liver metastasis or not. They concluded that despite having liver metastases and the possibility of experiencing a smaller benefit, hepatic metastases were not substantially related to the efficacy of the PD‐(L)1 inhibitor combined with chemotherapy as a first‐line treatment. According to the findings, the patients whether having hepatic metastasis or not, may benefit similarly from the treatment [172].

#### 4.6.3 Prostate Cancer

It is the second most prevalent cancer in the world and it is the fifth most common cause of cancer‐related mortality in men [173]. It is also the most frequent malignancy among men in 84 nations, with the industrialized world having the highest prevalence [174].

While blocking immune checkpoints has boosted responses and altered therapy paradigms in cancers like melanoma and NSCLC, effectiveness in prostate cancer remains low. In AR‐V7‐expressing metastatic prostate cancer, a regimen of ipilimumab alongside nivolumab has shown moderate effectiveness [175, 176, 177]. In patients suffering metastatic castration‐resistant prostate cancer (mCRPC), a lot of anti‐PD‐1 antibodies are now being tested in clinical studies. Cemiplimab is an anti‐PD‐1 antibody that has been authorized for the management of CSCC [178]. Cemiplimab is now being tested in combination with other treatments for prostate cancer. For example, NCT03367819 is a completed clinical trial evaluating isatuximab plus cemiplimab in prostate cancer and NSCLC [179].

According to a review by Bansal et al. [180] about mCRPC treatment with ICI monotherapy and combination therapy, we can be optimistic that ICIs might be effective in the future. A similar statement can be found in another study by Chen et al. [181] that although none of the monotherapy immunotherapies are anticipated to make a significant difference in prostate cancer consequences, but combination therapies may become more promising in the future.

#### 4.6.4 Colorectal Cancer

CRC, often termed as bowel cancer, colon cancer, or rectal cancer, is a kind of carcinoma that affects the intestines. It normally begins as a benign growth, frequently in the shape of a polyp, and progresses to a malignant status over time [182]. CRCs’ 5‐year survival rate is nearly 65% in the US [183].

Single‐agent anti‐PD‐1 antibodies are beneficial in treating metastatic CRCs and might be a potential first‐line therapeutic choice for patients suffering the dMMR/MSI‐H type of this malignancy. More reliable research on the use of these antibodies in the treatment of CRC patients is necessary as further evidence is required to make a more informed decision. He et al. [184] found that CRC patients who previously received therapy show a substantial increase in survival. Anti‐PD‐1 inhibitors are currently mainly used as a second or third line of treatment for CRC.

Another meta‐analysis has revealed that ICI‑related adverse events were not statistically different from the traditional treatments; but unfortunately, in advanced CRC patients, ICI treatment had no obvious advantage over the standard therapies [185]. However, we must consider that this systematic review has conducted the study with the limited data of 3 RCTs.

Furthermore it should be stated that despite ICIs’ impact on dMMR/MSI CRC treatment, unimpressive outcomes have been revealed in mismatch repair–proficient/microsatellite‐stable (pMMR/MSS) CRC patients' treatment [184, 185].

#### 4.6.5 Melanoma

Melanoma is the fifth most prevalent malignancy in the United States, and is also among the most aggressive types of skin cancer [186].

In 2022, China (excluding Taiwan provinces) and the United States (excluding dependencies) are expected to have 8114 and 99,935 new skin melanoma cases, sequentially. In China and the United States, mortality from melanoma of the skin are estimated to be 4369 and 7530, respectively [187].

The majority of early stage melanomas (i.e., Stages I and II) are treatable, with a 99.4% and 68% 5‐year survival rate, respectively [188]. Advanced melanoma (Stages III and IV) with dacarbazine treatment, on the other hand, had a disappointing 3‐year survival rate of 12.2% [189].

Almost all of the individuals with early‐stage melanoma benefit from surgical excision. Unfortunately, there have been limited therapy choices for individuals with unresectable melanoma or who acquire distant metastatic melanoma (e.g., unresectable stage III or Stage IV or advanced melanoma) [190]. Nowadays, there are many options for melanoma treatment. Some of these options are well reviewed by Carlino et al. [191].

Promisingly, there have been some ICI treatments approved for melanoma. Studies have revealed the optimal ICI doses for advanced melanoma management. It has been suggested that pembrolizumab, 2 mg/kg every 3 weeks and 10 mg per kg every 3 weeks; and nivolumab, 3 mg/kg every 2 weeks, could be the best therapy regimens (considering immune‐related adverse events risks) between several ICI regimen options. Whilst, monotherapy with ipilimumab, 10 mg/kg every 3 weeks, and combination therapy with nivolumab, 1 mg/kg every 3 weeks plus ipilimumab, 3 mg/kg every 3 weeks should be used carefully [192].

Despite significant advances in melanoma management, two patients’ melanoma out of every three patients will progress following ICI treatment, and 50% of the patients suffering advanced stages will pass away [191].

Besides the approved treatments for melanoma, we should consider studies addressing ICIs’ effectiveness in managing individuals with ocular melanoma [193].

A systematic review in brazil published in 2021 has revealed that as mAbs are so expensive in low and middle‐income nations, there is no justification of their advantages when it comes to replacing conventional medication in public health services [194]. On the other hand, in terms of being cost‐effective, another study in china suggests pembrolizumab, a more cost‐effective treatment, as a non‐first‐line drug for managing unresectable/metastatic melanoma compared to chemotherapy [195].

## 5 Environmental, Dietary, and Lifestyle Aspects in Immunotherapy

Emerging data shows that immunotherapy's success can be substantially affected by elements outside of basic tumor genetics and immune checkpoint pathways. Particularly, changes in the gut microbiome have been linked to the host's immunological response to ICIs. For patients with melanoma and NSCLC, for instance, higher abundance of beneficial bacterial species like *Akkermansia muciniphila* has been linked to better clinical outcomes [196, 197, 198].

Furthermore, environmental exposures, dietary habits, medications, and lifestyle choices, which all play important roles as “exposomes,” have been shown to influence systemic immune function [199]. Diets high in polyphenols and fiber can help maintain a good microbial ecology and lower systemic inflammation, hence improving the antitumor immune response. In addition, lifestyle choices like consistent exercise and stress management strategies support the immune system's performance [200, 201].

## 6 Molecular Pathological Epidemiology (MPE) in Immunotherapy

Molecular, genetic, metabolic, proteomic, and clinical data are combined with exposome elements, forming the discipline of MPE [202]. MPE provides a framework for evaluating how environmental, dietary, and lifestyle exposures interact with molecular changes in malignancies, therefore enabling the identification of predictive biomarkers and the creation of individualized treatment plans [203, 204, 205].

MPE combines molecular tumor profiling and epidemiological data to reveal heterogeneity in immunotherapy responses, as proven by The Cancer Genome Atlas Program (TCGA) and the Immuno‐Oncology‐Atlas Project [205, 206, 207]. In CRC, diet and micronutrients including vitamin D and marine omega‐3 fatty acids affect immune infiltration [208]. Environmental exposures including PM2.5 and aromatic amines change immune checkpoint expression patterns and lower treatment efficacy [205, 206]. Furthermore, PD‐1 inhibitor responses are modulated by dietary patterns and microbial metabolites, such as the consumption of processed meat versus high‐fiber diets which contain *Faecalibacterium prausnitzii* [204, 208]. Exercise and night shift work among other lifestyle choices influence circadian gene methylation and cytokine release, which influences treatment effects [203, 205, 209, 210]. Resistance and survival correlate with tumor microenvironment characterizations, including FAP‐positive cancer‐associated fibroblasts and endothelial‐immune cell dynamics [203, 209]. Ultimately, MPE focuses on clonal neoantigen evolution to maximize individualized immunotherapy and addresses ethical discrepancies in genetic testing [205, 211].

## 7 Conclusion

Over the past decade, as the immune checkpoint signaling pathways have been more thoroughly discovered, treatment with ICIs has been considered as a successful cancer treatment method. ICIs have gained more attention in the care of patients with a variety of advanced solid tumors. Anti PD‐1, anti PD‐L1, and anti CTLA‐4 immunotherapies have shown the most promising outcomes in the treatment of various malignancies including breast cancer, lung cancer, CRC, and melanoma. With these novel treatments and the combination therapies they offer, there is also hope for better treatment outcomes for the previously untreatable metastatic cancers. In spite of the beneficial aspects of immune checkpoint therapy, similar to other treatments, they may cause side effects in some patients. Therefore, more studies are needed to reduce the probable side effects and uncover their underlying mechanism. Based on the data shown in this review, there is still a lack of knowledge about the complete properties of ICIs and the possible combination therapies that we may be able to implement to achieve a better treatment response in the patients with cancer. It is hoped that through more comprehensive investigations about the immune checkpoint pathways, the discovery of new immune cascades, and a better understanding of the underlying evading mechanisms, new ICIs and other immune‐based medications can be designed and utilized in the future.

## Author Contributions

E.R.G. and R.M. conducted the literature review, and provided the initial draft. E.R.G. designed the figure. R.M. designed the table. P.L. and T.K. provided the idea of the research and revised the manuscript. A.S. critically revised and edited the manuscript. All the authors have contributed substantially to this study and have read and approved the final version of the article.

## Ethics Statement

The research protocol was approved by Student Research Committee.

## Conflicts of Interest

The authors declare no conflicts of interest.

## Data Availability

This article is a review article, and all of the information in the article is directly from available online sources and publications which are accessible in the reference section.
